# Excessive daytime sleepiness assessed by the Epworth Sleepiness Scale and its association with health related quality of life: a population-based study in China

**DOI:** 10.1186/1471-2458-12-849

**Published:** 2012-10-08

**Authors:** Shunquan Wu, Rui Wang, Xiuqiang Ma, Yanfang Zhao, Xiaoyan Yan, Jia He

**Affiliations:** 1 Department of Health Statistics, Second Military Medical University, 800 of XiangYin Road, Shanghai 200433, China

**Keywords:** Excessive daytime sleepiness, Mandarin, Epworth Sleepiness Scale, Health-related quality of life

## Abstract

**Background:**

Excessive daytime sleepiness (EDS) is a common condition worldwide that has many negative effects on people who were afflicted with it, especially on their health-related quality of life (HRQOL). The Epworth Sleepiness Scale (ESS) is a commonly used method for evaluating EDS in English-speaking countries. This paper reported the prevalence of subjective EDS in China as assessed by the Mandarin version of the ESS; tested the scale’s response rate, reliability and validity; and investigated the relationship between ESS scores and HRQOL.

**Methods:**

A population-based sample of 3600 residents was selected randomly in five cities in China. The demographic information was collected, subjective EDS was assessed by the Mandarin version of the ESS (ESS scores >10), and HRQOL was evaluated by the Mandarin version of the 36-item Short Form Health Survey (SF-36).

**Results:**

The Mandarin version of ESS had very few missing responses, and the average response rate of its eight items was 97.92%. The split-half reliability coefficient and Cronbach’s α coefficient were 0.81 and 0.80, respectively. One factor was identified by factor analysis with an eigenvalue of 2.78. The ESS scores showed positive skewness in the selected sample, with a median (Q1, Q3) of 6 (3, 0). 644 (22.16%) respondents reported subjective EDS, and all of the scores of the eight dimensions of the SF-36 were negatively correlated with ESS scores.

**Conclusions:**

The Mandarin version of ESS is an acceptable, reliable, and valid tool for measuring EDS. In addition, subjective EDS is common in China, based on the ESS results, and impairs HRQOL.

## Background

Excessive daytime sleepiness (EDS) is a public health concern. It can be caused by disorders such as obstructive sleep apnea (OSA), narcolepsy, and idiopathic hypersomnia [1-4]. A previous study showed that the prevalence of EDS was 2.5% in the Japanese general population [5], and another study showed an EDS prevalence of 8.7% in the general population of the United States [6]. Thus, EDS is common worldwide. People who suffer from EDS sleep a lot during the daytime and usually have cognitive and memory problems. In addition, EDS might be associated with impaired health-related quality of life (HRQOL) in patients with OSA [7] and narcolepsy [8-11]. However, there is a lack of data on the relationship between HRQOL and EDS. Briones et al. found that EDS had an important impact on general health and functional status, which they interpreted as reflecting HRQOL, as measured by the Medical Outcomes Study Short Form 36 [12]. Similar results were also obtained in another study [13].

The Epworth Sleepiness Scale (ESS) is a widely used subjective method for assessing EDS in English-speaking countries. Compared with objective methods such as the Multiple Sleep Latency Test (MSLT) and the Maintenance of Wakefulness Test (MWT), the ESS is simpler, cheaper, and less time-consuming [14]. Furthermore, it attempts to measure the general level of daytime sleepiness over a recent period, rather than providing information on an individual’s sleepiness at a particular time only [1]. The ESS is an eight-item, self-administered questionnaire that contains eight situations commonly encountered in daily life. The subject is instructed to answer how likely it is that he/she would fall asleep in those different situations, by giving a score from 0 to 3. Thus, the total score of the ESS ranges from 0 to 24. The higher the score, the greater the possibility the individual will fall asleep during the daytime.

The English version of the ESS has been demonstrated to have high reliability and consistency [15]. Different versions of the ESS have also been developed in non-English speaking countries [16-20]. The Korean version has good internal consistency (Cronbach’s α coefficient = 0.90) and test-retest reliability (r = 0.78–0.93) [19]. In the Japanese version of the ESS, the response rate is usually quite high with the percentage of missing values ranging from 0.7% to 1.1%, and the Cronbach’s α coefficient is 0.85. The test-retest reliability of the Japanese version is also quite high (r = 0.78) [20]. The ESS has been used extensively in sleep-disordered and medical populations [21-24]. However, seldom have studies focused on a Mandarin version of the ESS, and few studies have used the ESS to assess the general population.

In this study, we aimed to (1) evaluate the response rate, reliability, and validity of the Mandarin version of ESS; (2) assess the prevalence of subjective EDS among the Chinese general population by using the ESS; and (3) assess the relationship between ESS scores and HRQOL.

## Methods

### Study design and sample

This study was part of a countrywide survey, and the details of the methodology of this survey were published elsewhere [25]. From April 2007 to January 2008, a population-based sample of 3600 individuals aged 18 to 80 years was selected randomly in five cities of China—Shanghai, Beijng, Xi’an, Wuhan, and Guangzhou—using a multiple-stage sampling method with stratification according to the proportion of the population in a particular town of a particular age and sex, as reported by the local government. We obtained the basic demographic information of residents from local residential committee offices before conducting this survey. Residents who were illiterate, not in the 18–80 age group, or suffering from psychiatric illnesses or other disabilities were excluded from the study. Respondents completed the questionnaires by themselves in their household or in local resident committee offices. All gave written informed consent before participation. The study was approved by the Second Military Medical University Ethics Committee in Shanghai, China.

### Measurements

We gathered sociodemographic information, including region of residence, gender, age, height, weight, educational level, and occupation, and data on the prevalence of chronic diseases, including hypertension, diabetes, cerebrovascular diseases, liver diseases, renal diseases, chronic bronchitis, and rheumatoid arthritis.

**Figure 1:**
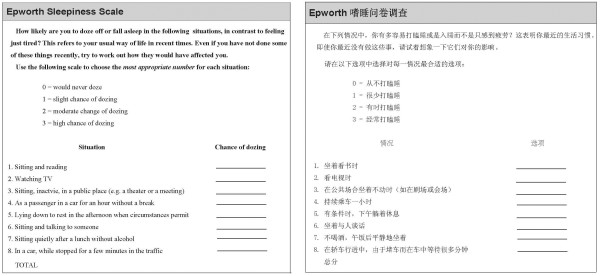
The English version of the Epworth Sleepiness Scale and the Mandarin version of the Epworth Sleepiness Scale.

The Mandarin version of the ESS was used to evaluate EDS. It is an eight-item, self-administered questionnaire, with a total score ranging between 0 and 24. A score was greater than 10 indicates subjective EDS [1,21]. The English ESS was translated into Chinese through a three stage process. First, two postgraduates of public health independently translated the original ESS into Mandarin. Both translators had experience in translating questionnaires but were not familiar with the ESS. Second, two English teachers translated the Mandarin version of the ESS back into English. Disagreements were discussed among experts on epidemiology and public health, and then we developed a revised version. Finally, the revised version was tested among literate volunteers and some minor changes were made to develop a final version. The English and Mandarin versions of ESS are shown in Figure 1.

Furthermore, we assessed the respondents’ HRQOL by using the Mandarin version of SF-36, a generic HRQOL instruments that was translated from the International Quality of Life Assessment (IQOLA) SF-36 Standard UK Version 1.0 by experts in Zhejiang University, who also tested its reliability and validity [26]. It comprises 36 questions divided into eight dimensions: physical functioning (PF), role limitation due to physical problems (RP), role limitation due to emotional problems(RE), social functioning (SF), mental health (MH), vitality (VT), bodily pain (BP), and general health perception (GH) [27].

### Statistical analysis

Independent double data entries were conducted by two professional data managers using the EpiData 3.1 software. Both manual and computer checking were used to locate any discrepancies. Statistical Analysis System (SAS) 9.1.3 was used to analyze survey data. All hypothesis tests used two-sided tests, and p-values of less than 0.05 were considered statistically significant.

The ESS was assessed in terms of response rate, reliability, and validity. Response rate was calculated with respect to the missing values and total values. Split-half reliability was computed by correlating the scores of the odd half of the scale with those of the even half. Internal consistency was assessed using Cronbach’s α coefficient [28]. A Cronbach’s α of 0.7 or higher is generally considered sufficient to demonstrate internal consistency. Construct validity was assessed using correlational analysis and principal factor analysis with the prior communality estimate for each variable set to its squared multiple correlation with all other variables.

Both the Wilcoxon rank sum test and the Kruskal-Wallis *H* test were used to analyze the ESS scores among different groups. The correlation between ESS scores and SF-36 dimensions was assessed using Spearman’s correlation coefficient, and multiple linear regression analysis was conducted to control for sociodemographic variables. SF-36 scores of the EDS and normal groups were compared using an analysis of variance.

## Results

### Demographic information and response rate

Of the 3600 respondents, 3219 completed the self-administered questionnaire, with a total response rate of 89.42%; data from five were excluded from the analysis because of logical errors or insufficiently completed questionnaires, leaving a total of 3214 respondents with data suitable for analysis. Among the 3214, 192 (5.97%) missed one ESS item, and 116 (3.61%) missed no less than two items; 2906 (90.42%) respondents answered all eight ESS items, and these respondents’ total ESS scores were calculated and used to assess prevalence of subjective EDS. The average response rate of the eight items was 97.92%, ranging from 94.12% (Item 1) to 99.69% (Item 6; Table 1). Logistic regression showed associations between the missing items and region, age, education, occupation, and prevalence of chronic diseases. Response rates were higher for urban than rural areas (*p* < 0.01). Response rates significantly decreased for respondents over 50 (*p* < 0.01), but significantly increased as the level of education improved (*p* < 0.01); similarly, more manual workers than office workers had missing items (*p* = 0.01). Respondents with chronic diseases had more missing data (*p* < 0.01). Missing data among different sociodemographic variables are shown in Table 2.

**Table 1:** Item responses, item scores, reliability and validity of ESS

**Item**	**Response**		**Item score**		**Cronbach's α**	**Factor analysis**
	***n***	**%**	**Median (Q1,Q3)**	**Mean (SD)**		
ESS1	3025	94.12	1(0,2)	0.99(1.09)	0.78*	0.57
ESS2	3187	99.16	1(0,2)	0.99(1.07)	0.78*	0.55
ESS3	3169	98.60	0(0,1)	0.70(0.94)	0.77*	0.68
ESS4	3174	98.76	1(0,2)	0.96(1.08)	0.76*	0.69
ESS5	3143	97.79	2(0,2)	1.48(1.14)	0.79*	0.53
ESS6	3204	99.69	0(0,0)	0.15(0.48)	0.80*	0.44
ESS7	3190	99.25	0(0,2)	0.86(1.03)	0.77*	0.61
ESS8	3086	96.02	0(0,1)	0.69(0.97)	0.78*	0.61
Total	2906	90.42	6(3,10)	6.75(5.11)	0.80	

* Cronbach's α,if the item was deleted.

**Table 2:** Characteristics of the sample, missing data, ESS scores and analysis of subjects with EDS

**Characteristic**	**Subjects [*n*(%)]**	**Subjects with missing data*n*(%)**	**Total ESS Score (*n*=2906)**		**Subjects with EDS (ESS>10)**		
					***n*(%)**	**Multivariate analysis**	
			**Median (Q1,Q3)**	**Mean (SD)**		**Odds ratio (95%CI)**	***p*-value**
Region							
Urban	1585(49.32)	41(2.59)	6.00(3.00,10.00)	6.74(4.93)	339(21.96)	1.00	
Rural	1629(50.68)	267(16.39)	6.00(2.00,10.00)	6.77(5.31)	305(22.39)	1.05(0.86,1.27)	0.65
Gender							
Female	1678(52.21)	175(10.43)	6.00(2.00,10.00)	6.73(5.17)	336(22.36)	1.00	
Male	1536(47.79)	133(8.66)	6.00(3.00,10.00)	6.77(5.05)	308(21.95)	0.97(0.81,1.17)	0.77
Age(years)							
18~29	749(23.30)	32(4.27)	6.00(4.00,10.00)	7.03(4.64)	161(22.45)	1.00	
30~39	736(22.90)	41(5.57)	6.00(3.00,10.00)	6.8(4.95)	153(22.01)	0.94(0.72,1.22)	0.63
40~49	757(23.55)	69(9.11)	6.00(2.00,10.00)	6.43(5.23)	148(21.51)	0.90(0.68,1.19)	0.47
50~59	486(15.12)	62(12.76)	6.00(2.00,9.50)	6.4(5.24)	83(19.58)	0.71(0.51,0.98)	0.04
60~80	486(15.12)	104(21.40)	6.00(2.00,11.00)	7.1(5.82)	99(25.92)	0.80(0.56,1.15)	0.23
Education							
Primary school or lower	571(17.77)	147(25.74)	7.00(3.00,11.00)	7.33(5.78)	124(29.25)	1.00	
Secondary/high school	1993(62.01)	152(7.63)	6.00(3.00,9.00)	6.43(4.95)	348(18.90)	0.57(0.44,0.76)	<0.01
University or higher	650(20.22)	9(1.38)	7.00(3.00,11.00)	7.31(5.01)	172(26.83)	0.95(0.67,1.35)	0.77
Occupation							
Office worker	912(28.44)	27(2.96)	6.00(3.00,10.00)	6.72(4.81)	197(22.26)	1.00	
Manual worker	2295(71.56)	281(12.24)	6.00(3.00,10.00)	6.76(5.25)	444(22.05)	1.07(0.86,1.34)	0.55
BMI							
18.5-23.9	1872(58.35)	153(8.17)	6.00(3.00,10.00)	6.44(4.92)	348(20.24)	1.00	
<18.5	296(9.23)	35(11.82)	7.00(4.00,10.00)	7.15(4.88)	61(23.37)	1.12(0.81,1.54)	0.50
24.0-27.9	810(25.25)	94(11.60)	7.00(3.00,10.00)	7.17(5.41)	173(24.16)	1.23(0.99,1.53)	0.06
>=28.0	230(7.17)	25(10.87)	7.00(3.00,12.00)	7.34(5.57)	62(30.24)	1.64(1.18,2.29)	<0.01
Chronic diseases*							
No	2382(74.11)	186(7.81%)	6.00(3.00,10.00)	6.64(4.97)	455(20.72)	1.00	
Yes	832(25.89)	122(14.66)	6.00(3.00,11.00)	7.08(5.52)	189(26.62)	1.36(1.08, 1.70)	<0.01

*Chronic diseases: including hypertension, diabetes, cerebrovascular diseases, liver diseases, renal diseases, chronic bronchitis, and rheumatoid arthritis.

The demographic characteristics of the survey respondents have been described in detail elsewhere [25]. Among the 3214 respondents, 52.21% were female and 47.79% were male. The average age was 42 years.

### Reliability

The split-half reliability coefficient and Cronbach’s α coefficient of the Mandarin version of the ESS were 0.81 and 0.80, respectively. The Cronbach’s α coefficients were calculated eight times, each time eliminating one of the eight items, and these values ranged from 0.76 to 0.80 (Table 1). The removal of specific items did not substantially increase internal consistency, which indicated that none of the items had an unusually strong influence on internal consistency.

### Validity

Significant Spearman’s correlations were found between each item score and the total score, with coefficients all higher than 0.6 except in Item 6, which had a coefficient of 0.39 (*p* < 0.001). Each item significantly correlated with all other items (*p* < 0.001). Items 4 and 8 had the highest correlation, with a Spearman’s correlation coefficient of 0.60.

Factor analysis yielded only one factor with an eigenvalue of 2.78, and the other eigenvalues were below 0.50. Factor loadings for Items 1–8 were 0.57, 0.55, 0.68, 0.69, 0.53, 0.44, 0.61 and 0.61, respectively (Table 1).

### ESS score

**Figure 2:**
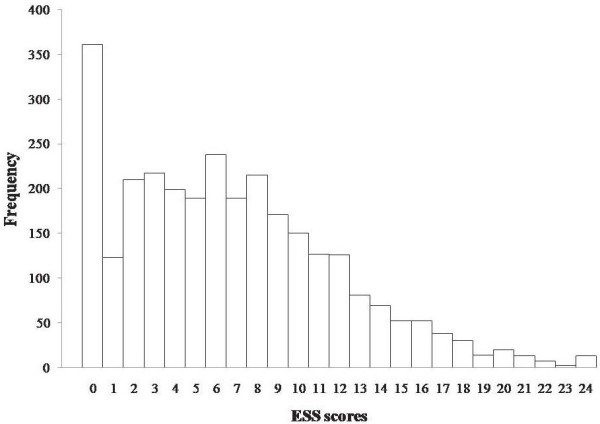
Distribution of ESS scores in the sample.

The ESS total score distribution of the selected sample (n = 2906) is shown in Figure 2. The data were not normally distributed, but showed positive skewness. A large proportion of the respondents (361; 12.42%) had a total score of zero. The median (Q1, Q3) of the ESS scores was 6 (3, 10), and the mean (SD) was 6.75 (5.11).

No significant differences in ESS scores were found among different regions, genders, ages, and occupation groups, while education and BMI were two notable factors related to EDS. Compared with those with a higher level of education and those with a lower level of education, respondents with a medium level of education had lower ESS scores, indicating less EDS. Respondents with normal BMI level (18.5–23.9) had lower ESS scores compared with those whose BMIs were above or below this level. The median (Q1, Q3) and mean (SD) of ESS scores among the different variables are shown in Table 2.

Item analysis showed that Item 5 received the highest score, with a median (Q1, Q3) of 2 (0, 2) and a mean (SD) of 1.48 (1.14). Item 6 received the lowest score, with a median (Q1, Q3) of 0 (0, 0), which meant that at least 75% of respondents gave that item a score of 0, and a mean (SD) of 0.15 (0.48) (Table 1).

### Prevalence of EDS

Among the 2906 respondents whose total ESS scores had been calculated, 644 reported subjective EDS, with a prevalence of 22.16% (95% confidence interval: 20.65%–23.67%). Prevalences of EDS in different subgroups and the associated sociodemographic factors are shown in Table 2. Among all the factors, education, BMI, and prevalence of chronic diseases were significantly associated with the prevalence of EDS. EDS prevalence was lower in respondents with a secondary or high school education, compared with those who had education levels of only primary school or below (*p* < 0.01). However, this trend was not seen among respondents with education levels of university or higher. EDS was more prevalent in respondents with BMIs of 28.0 and above than in those with normal BMIs (18.5–23.9) (p < 0.01). The prevalence of EDS was significantly higher among respondents with chronic diseases than in those not suffering from such diseases (p < 0.01).

### HRQOL

ESS scores were significantly negatively correlated with the scores on the PF, RP, BP, GH, VT, SF, RE, and MH subscales of the SF-36 (*p* < 0.001); the Spearman’s correlation coefficients were −0.12, -0.12, -0.14, -0.23, -0.24, -0.15, -0.13, and −0.23, respectively. The total SF-36 scores were also significantly negatively correlated with ESS scores, with a Spearman’s correlation coefficient of −0.28 (*p* < 0.001). After further consideration of the sociodemographic variables, including region, gender, age, education, occupation, BMI, and prevalence of chronic diseases, using multiple linear regression analysis, we found that the scores of the PF, RP, BP, GH, VT, SF, RE, and MH scales were still significantly negatively correlated with ESS scores (*p* < 0.001), with the standard regression coefficients being −0.13, -0.12, -0.13, -0.24, -0.25, -0.16, -0.13, and −0.22, respectively.

**Figure 3:**
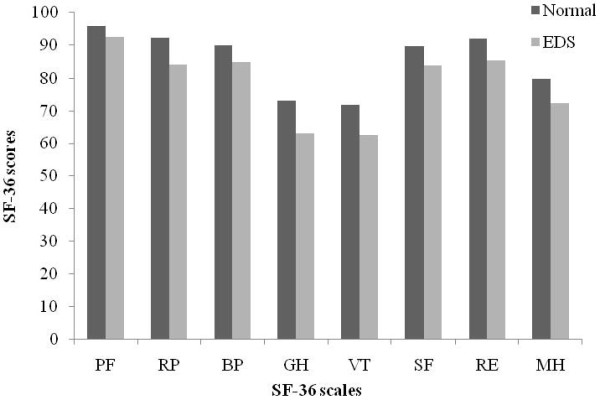
**Comparisons of SF-36 scores between EDS group and normal group.***p*<0.001. *PF*: physical functioning; *RP*: role limitation due to physical problems; *BP*: bodily pain; *GH*: general health perception; *VT*: vitality; *SF*: social functioning; *RE*: role limitation due to emotional problems; *MH*: mental health.

We also compared HRQOL between normal respondents and respondents with EDS (ESS scores > 10), and found that the respondents with EDS had lower scores in all SF-36 scales than the normal respondents (*p* < 0.001) (Figure 3).

## Discussion

EDS is a typical symptom of sleep disorders such as OSA, narcolepsy, and idiopathic hypersomnia. Studies have reported that 2% of women and 4% of men in the general middle-aged population suffer from OSA [29,30]. ESS has proven to be a useful tool in the assessment of EDS in English and some non-English-speaking countries. Our study reported the response rate, reliability, and validity of the Mandarin version of the ESS when applying it to the general population in five cities of China.

We found that 90.42% of the respondents answered every ESS item, indicating that the Mandarin version of the ESS was acceptable to the majority of the population. In addition, the different response rates among some associate factors were explicable: Response rates were higher for urban than rural areas because, as a whole, the urban respondents were more educated than rural ones. Additionally, rural respondents had fewer opportunities to encounter some items in their daily life such as Items 4 and 8. For the elderly, due to their physical and intellectual limitations, it was difficult to complete the questionnaires without others’ help. Consequently, the response rate of the elderly was lower than that of the young. The fact that more educated respondents had a higher response rate was quite natural, as they had greater knowledge and more social responsibilities. Office workers engaged more often in mental work, and likely had better comprehension and more energy to complete the questionnaires than manual workers. Thus, the response rate of office workers was higher than that of manual workers. Finally, it is understandable that respondents with chronic diseases had more missing data, as their physical condition might limit their ability to complete the scale.

The response rate of each item was mainly determined by how frequently the situation was encountered in daily life. Sitting and talking was very common every day, so Item 6 had the highest response rate. In contrast, sitting and reading was seldom encountered in some populations like the elderly, rural residents, the less educated, and manual workers, so Item 1 had the lowest response rate.

The split-half reliability coefficient was 0.81. The internal consistency of the questionnaire was fair, with a Cronbach’s α coefficient of 0.80, which was similar to what has been reported previously (0.74–0.90) [1,15,19,21,31,32]. This consistency did not increase when any of the items were excluded. All of these values indicated excellent reliability and internal consistency [18]. Each item score significantly correlated with total score and all other item scores. In addition, the results of the factor analysis showed that only one factor was extracted with an eigenvalue greater than one. Hence, this questionnaire was thought to have good construct validity. All of these results suggest that the ESS is suitable for the Chinese population despite the differences in language and cultural background.

The mean (SD) of the ESS scores among the 2906 respondents who answered all eight ESS items was 6.75 (5.11) in our study. In the Turkish version of the ESS, the mean (SD) ESS score was 3.6 (3) among 60 healthy controls and 12.6 (6) among 60 respondents with sleep-disordered breathing [18]; in the Korean version, the mean (SD) ESS score was 5.07 (2.93) among 60 healthy controls and 8.21 (4.23) among 213 patients [19]. In most previous studies, the ESS scores were observed from the healthy controls vs. patients with sleep-disordered diseases, using a small population sample, while in our study, the ESS scores were observed from a large general population sample. We thought that it was also important to apply the ESS to the general population. Our results indicated that 22.16% of respondents had an ESS score above 10. According to the original version of the questionnaire, a score of 10 is considered the cut-off point that distinguishes normal respondents from patients with EDS [1,21]. Nearly a quarter of respondents in the selected sample had subjective EDS according to this standard. Previous studies conducted in Japan showed that 2.5% of the Japanese general population and 15% of the Japanese general adult population had EDS, as assessed by other questionnaires [5,33]. Another study reported that the prevalence of EDS as assessed by the ESS (ESS scores > 10) was 12.2% among the adult population of Korea [34]. From these and our findings, we could say that EDS is more common in China than in these two East Asian countries. However, the basis of this upper limit of normal sleepiness needs to be further investigated. The more widespread prevalence of EDS in China may be due to dramatic increases in obesity and overweight in Mainland China, especially in metropolises such as Beijing and Shanghai [35]. The extensive use of electronic products, such as mobile phones, computers, and television, has also likely contributed to the high prevalence of EDS [36]. Moreover, there is a cultural peculiarity of China in that many Chinese people tend to take afternoon naps, which might make respondents give higher ESS scores. In addition, we found an interesting correlation between education and ESS scores. People with a low level or a high level of education had higher ESS scores, compared with those with a medium level of education. We propose the following explanations for this phenomenon: First, a higher proportion of people with a low level of education are unemployed; second, people with a high level of education are mainly brainworkers and insomnia is one of the diseases exceedingly common among this group. Thus, people with lower or higher levels of education are more prone to sleep during the daytime. We also found that EDS was more common among respondents with obesity and other chronic diseases, a finding consistent with those of previous studies [37,38].

Item analysis of the Mandarin version of the ESS revealed results that were very close to those reported for the original version [15,39]. Item 5 was the most soporific and got the highest score, while the scores for Items 6 and 8 were very low. This means when “lying down to rest in the afternoon when circumstances permit,” respondents were most likely to fall asleep, while when “sitting and talking to someone” or “in a car while stopped for a few minutes in the traffic,” they were less likely to sleep.

Two other Chinese versions of the ESS have been developed, in Hong Kong [40] and Taiwan [41]. However, these are in traditional Chinese, while the current version is in simplified Chinese. Furthermore, the lifestyles and economic backgrounds of the populations in these two regions differ from those in Mainland China. Zhang et al. [42] and Peng et al. [43] have also developed two simplified Chinese versions of the ESS. In the former study, the researchers created a modified ESS by adding two backup items to the original ESS and forming a ten-item Sleepiness Questionnaire; however, this practice might have changed the original meaning of the scale. Zhang et al. found that the modified ESS was not reliable for normal respondents, though they mainly applied it among patients with suspected sleep-disordered breathing. In Peng et al.’s study, the translations of the items were not the same as those in our study. For example, in Item 8, “in a car” was translated as in their study as “when driving,” and “while stopped for a few minutes in the traffic” was translated as “while waiting for the traffic lights or when stopped in traffic for a few minutes.” Obviously, this would lead the respondents to give a lower item score, because people are less likely to sleep when they are a driver rather than a passenger, and when they are waiting for the traffic lights to change, which is a rather short time, instead of waiting in a traffic jam. In addition, the respondents in their study were patients with suspected obstructive sleep apnea hypopnea syndrome. The strength of our study is that we made the translation strictly in accordance with the meaning of the original version and then applied it to the general population.

The correlations between ESS scores and the scores of the SF-36 dimensions indicated that the eight dimensions of the SF-36 negatively correlated with ESS scores, even after controlling for the sociodemographic variables. SF-36 scores significantly decreased as ESS scores increased. The scores of the GH and VT scales decreased the most. EDS influences more or less all parts of life to such an extent that people with the condition perceive themselves as being generally more limited by their health than those without it. They are more likely to worry about their future and whether the symptoms will improve, leading to increasing dependency on family and friends [44]. The VT scale was the second most affected dimension in this study. People with EDS experience ineluctable sleep episodes. These symptoms may lead to the feeling of physical tiredness, causing reduced vitality and energy [9]. These results were consistent with those reported in other countries [45,46].

Therefore, we concluded that the Mandarin version of the ESS was acceptable and applicable among the general population of five cities in China. As measured by the ESS, subjective EDS is common in China. Special care should be taken for those people who suffer from EDS, as it can disrupt a person’s social life and threaten public health and safety [47-49]. We propose that the Mandarin version of the ESS can be used as an auxiliary judgment of EDS, and that early detection and treatment after the onset of symptoms might be important in reducing the negative effects on the quality of life of the patients.

There were four limitations in our study. First, we did not contact the original developer of the ESS, which might lead to a lack of conceptual equivalence with the original version. Second, this was only a general population-based study, and the Mandarin version of the ESS was not assessed using patients diagnosed with EDS; thus, the response rate, reliability, and validity of the Mandarin version among the patient population are uncertain. Furthermore, some items, which can be used as the criteria for known-groups validity such as sleep-wake patterns, symptoms and history of sleep disorders, using sleep pills, sleep quality (using the Pittsburgh Sleep Quality Index and/or other validated questionnaires), and so on, were not measured. Third, the ESS is only a subjective method that can be used as an auxiliary judgment of EDS diagnosis, but cannot act as a gold-standard test, so the prevalence of EDS found in this study might be overestimated. The diagnosis of EDS should be further validated by objective methods. Finally, the sample population was selected from five economically developed regions in China. Therefore, the situation in less developed regions was not investigated. To address these limitations, further investigation should be undertaken.

## Conclusion

In summary, the Mandarin version of the ESS is an acceptable, reliable, and valid questionnaire for screening EDS, and it could be used as an auxiliary judgment for an EDS diagnosis. EDS was common in the selected sample of the Chinese population, and people with lower or higher levels of education should be paid more attention, to identify the reasons for their increased prevalence. In addition, EDS is associated with the impairment of HRQOL, and this should be addressed by public health officials.

## Competing interests

Shunquan Wu, Rui Wang, Xiuqiang Ma, Yanfang Zhao and Xiaoyan Yan have no statement of interests. Jia He has served as the director of the Department of Health Statistics, Second Military Medical University.

## Authors' contributions

JH conceived of the study and supervised all aspects of its implementation. SW and R W assisted with the survey, completed the statistical analyses and led the writing of different versions of the manuscript. Y Z and X Y assisted with the study, and X M assisted with the survey and data analyses. All authors contributed to conceptualize ideas, interpret findings, and review the drafts of the manuscript. All authors read and approved the final manuscript.

## Pre-publication history

The pre-publication history for this paper can be accessed here:

http://www.biomedcentral.com/1471-2458/12/849/prepub
